# 通过型固相萃取-超高效液相色谱-串联质谱法同时测定中华鳖中8种抗病毒药物及2种代谢物

**DOI:** 10.3724/SP.J.1123.2024.02011

**Published:** 2024-12-08

**Authors:** Yaqin WANG, Kun WANG, Qingqing KE, Yuchong TAN, Dingnan WANG, Shiyan LI

**Affiliations:** 1.浙江大学生物技术研究所,水稻生物育种全国重点实验室,浙江 杭州 310058; 1. State Key Laboratory of Rice Biology and Breeding, Institute of Biotechnology, Zhejiang University, Hangzhou 310058, China; 2.浙江省水产技术推广总站,浙江 杭州 310023; 2. Zhejiang Fisheries Technology Extension Center, Hangzhou 310023, China

**Keywords:** 通过型固相萃取, 亲水作用色谱, 超高效液相色谱-串联质谱, 抗病毒药物, 代谢物, 中华鳖, pass-through solid-phase extraction, hydrophilic interaction chromatography (HIC), ultra-high performance liquid chromatography-tandem mass spectrometry (UHPLC-MS/MS), antiviral drugs, metabolites, Chinese softshell turtle

## Abstract

本研究以中华鳖中8种抗病毒药物(antiviral drugs, ATVs)及2种代谢物为研究对象,采用Wallelut Lipoclean磷脂去除小柱净化样品,建立了通过型固相萃取净化模式与超高效液相色谱-串联质谱法(UHPLC-MS/MS)相结合的快速检测方法。样品在37 ℃下经酸性磷酸酯酶酶解,1%乙酸乙腈提取,通过型固相萃取小柱净化,40 ℃下氮吹浓缩后采用UHPLC-MS/MS检测,多反应监测(MRM)模式采集数据,内标法定量。针对不同ATVs之间极性差异大的特点,实验优化了仪器参数和前处理条件,重点对色谱柱进行了筛选并排除了生物内源性物质尿苷对利巴韦林的分析干扰,同时讨论了前处理过程中提取液和净化小柱选择对提取效率和净化效果的影响。在最优实验条件下,10种目标物在一定质量浓度范围内线性关系良好,相关系数(*r*^2^)为0.994~0.998,方法检出限(MDL)为0.05~1.1 μg/kg,方法定量限(MQL)为0.18~3.8 μg/kg。在低、中、高3个添加水平下10种目标物的平均回收率范围为82.5%~103%,相对标准偏差(RSD)为3.11%~12.1%。该方法操作简便、快速、灵敏,重复性好,可用于大批量样品的同时快速检测,并成功应用于省级水产品质量安全风险监测工作。

## Abstract (en)

Antiviral drugs (ATVs) are not licensed for the treatment of viral diseases in food-producing animals in China. However, they are reportedly illegally used in the Chinese softshell turtle (*Pelodiscus sinensis*) aquaculture industry; hence, the use of ATVs has become a new aquatic food-safety problem. In this study, a method was developed for analyzing eight ATV residues (amantadine, rimantadine, lamivudine ribavirin, moroxydine, acyclovir, oseltamivir, and imiquimod) and two metabolites (1,2,4-triazole-3-carboxamide and oseltamivir acid) in Chinese softshell turtle muscle using pass-through solid-phase extraction and ultra-high performance liquid chromatography-tandem mass spectrometry (UHPLC-MS/MS). The main factors that affect the sensitivity, precision, and accuracy of the method were optimized during sample pretreatment and instrumental analysis owing to the diverse chemical properties of the analytes. The performance of various chromatographic columns and mobile phases were investigated to eliminate interference from uridine during the detection of ribavirin. The proportion of acetonitrile in extration solution and solid-phase extraction column type were prioritized when optimizing extraction and purification parameter. Samples were hydrolyzed using phosphatase (acid type) in 6 mL ammonium acetate buffer, with optimum results obtained when enzymolysis was performed at 37 ℃, and pH 4.8 for 2 h, after which they were extracted with 14 mL of acetonitrile containing 1% acetic acid. The supernatant (2 mL) was purified using a Wallelut Lipoclean column operated in pass-through solid-phase-extraction mode. The sample was dried under nitrogen at 40 ℃ and the residue was dissolved in 1 mL of methanol-acetonitrile (50∶50, v/v) solution. The sample was finally filtered through a 0.22-μm hydrophilic polytetrafluoroethylene syringe filter for UHPLC-MS/MS. The chromatographic separation of the analytes was performed on a hydrophilic interaction chromatographic column (HILIC-Z, 100 mm×2.1 mm, 2.7 μm) via gradient elution using 2 mmol/L ammonium acetate aqueous solution (containing 0.1% formic acid) and acetonitrile as mobile phases A and B, respectively. The linear gradient program were as follows: 0-1.0 min, 3%A; 1.0-5.0 min, 3%A-20%A; 5.0-6.0 min, 20%A; 6.0-6.5 min, 20%A-50%A; 6.5-8.0 min, 50%A; 8.0-8.5 min, 50%A-3%A; 8.5-12 min, 3%A. The analytes were detected using a triple quadrupole mass spectrometer operating in positive-ion scanning and multiple-reaction monitoring (MRM) modes, with quantitative analysis performed using the internal standard method. Good linearities (*r*^2^=0.994-0.998) were obtained for the ten target compounds at experimental concentrations under the optimal conditions. The method detection limits (MDLs) and method quantification limits (MQLs) were in the range of 0.05-1.1 and 0.18-3.8 μg/kg, respectively. The average recoveries of the 10 target compounds ranged from 82.5% to 103%, with relative standard deviations (RSDs) in the range of 3.11%-12.1% at low, medium and high spiked levels. The developed method was applied to 67 Chinese softshell turtle samples obtained from a fish farm in Zhejiang Province. Ribavirin and metabolite were detected in real samples at total contents of 6.02-333.2 μg/kg. The developed method is simple to operate, highly accurate, and suitable for use in risk-monitoring applications owing to its ability to rapidly determine eight ATV residues and two metabolites in Chinese softshell turtle samples.

抗病毒药物(antiviral drugs, ATVs)种类众多,至今已累计发现约100种^[1,2]^,其曾作为兽药被广泛用于动物疫病的防治^[3]^。考虑到食品安全与耐药性问题,我国农业部于2005年发布了第560号公告,明确禁止在农业养殖中使用金刚烷胺等ATVs^[4]^。当前,动物源性食品中ATVs监测主要关注金刚烷胺、金刚乙胺、拉米夫定、利巴韦林、吗啉胍、阿昔洛韦、奥司他韦、咪喹莫特等广谱类ATVs和抗流感、抗疱疹病毒类ATVs^[5⇓-7]^。由于部分ATVs在生物体内可以形成代谢物^[8,9]^,同时监测原药和代谢物对于ATVs的生物代谢机理和残留风险研究具有重要意义。中华鳖,俗称甲鱼,其味道鲜美又具有一定的药用价值,是我国重要的淡水渔业经济养殖品种^[10]^。随着中华鳖养殖集约化程度不断提高,其病毒性疾病的发生率也不断提升^[11,12]^,极易导致ATVs的滥用风险。此外,部分ATVs在水环境中可以长期稳定存在^[13,14]^,中华鳖等水生动物易受其暴露影响,造成ATVs的蓄积。目前,国内尚未有中华鳖中ATVs及代谢物残留的研究报道,相关风险值得关注。

近年来,超高效液相色谱-串联质谱法(UHPLC-MS/MS)因灵敏度高、抗基质干扰能力强,已成为动物源性食品中ATVs检测的主流分析方法^[15⇓-17]^。由于不同ATVs之间理化性质差异大,采用UHPLC-MS/MS建立的ATVs及代谢物高通量检测方法在兼顾方法灵敏度和准确度上仍存在着不小挑战。尿苷被报道是利巴韦林在质谱分析时的生物内源性干扰物,在建立利巴韦林的检测方法时需要排除其干扰^[18,19]^。不同种类ATVs及代谢物极性跨度较大,在不同种类色谱柱上保留能力和质谱响应也存在差异,吗啉胍等强极性ATVs在反相色谱柱上几乎不保留,质谱响应也普遍低于亲水作用色谱^[20]^,可见针对ATVs及代谢物的UHPLC-MS/MS方法对于仪器条件特别是色谱条件的优化有着更为严苛的要求。前处理方面,动物源性食品中ATVs净化通常采用吸附-洗脱模式固相萃取法^[6,21⇓-23]^,该方法的固相萃取小柱需要通过活化、上样、淋洗和洗脱4个步骤,操作繁琐。此外,单类别小柱目前难于满足多种类ATVs的高通量分析,如Berendsen等^[17]^为保证目标药物的回收率最终采用了双固相萃取小柱串联方式的净化方法,用于测定家禽肌肉中7种抗病毒药物残留。与此相比,QuEChERS法^[20,24]^和通过式固相萃取法^[25,26]^因操作简便已逐步应用于动物源性食品中ATVs的检测。特别是通过式固相萃取法在药物分析时可以同时兼顾检测效率和净化效果,在多种类药物分析上具有更为显著的优势^[27]^。

针对上述问题,本研究以养殖业中重点关注的ATVs为目标物,通过色谱柱筛选,优化色谱条件排除尿苷对利巴韦林分析的干扰,结合通过式固相萃取方法实现了杂质和目标物的一步式分离净化,建立了中华鳖中8种ATVs及2种代谢物的UHPLC-MS/MS高效分析技术,为中华鳖中ATVs残留风险研判提供了技术支持。

## 1 实验部分

### 1.1 仪器、试剂与材料

ExionLC AD-4500 Qtrap超高效液相色谱-串联质谱仪(美国SCIEX公司); Allegra X-12R高速冷冻离心机(美国Beckman公司); Multi Reax多位试管振荡器(德国Heidolph公司); Milli-Q超纯水仪(美国Millipore公司)。

金刚烷胺、金刚乙胺、1,2,4-三氮唑-3-甲酰胺、拉米夫定、利巴韦林、吗啉胍、阿昔洛韦、奥司他韦、奥司他韦酸、咪喹莫特标准品纯度均大于96%,购自天津阿尔塔科技有限公司;尿苷、金刚烷胺-D_6_、金刚乙胺-D_4_、利巴韦林-^13^C_5_、拉米夫定-^13^C,^15^N_2_、阿昔洛韦-D_4_、奥司他韦-D_3_、奥司他韦酸-D_3_、咪喹莫特-D_9_,纯度均大于92%,购自加拿大TRC公司;乙腈、甲醇、甲酸(色谱纯,美国Fisher公司);乙酸铵(色谱级,上海麦克林公司);酸性磷酸酯酶(活力>0.4 unit/mg,美国Sigma公司)。Wallelut Lipoclean磷脂去除小柱(300 mg/3 mL,深圳逗点生物技术有限公司)。

### 1.2 溶液配制

分别准确称取10 mg标准品于100 mL容量瓶中,用甲醇溶解并定容,配制成质量浓度为100 mg/L的标准储备液,冷冻储存;分别准确称取10 mg同位素内标药物于100 mL容量瓶中,用甲醇溶解并定容,配制成质量浓度为100 mg/L的同位素内标储备液,冷冻储存。用甲醇稀释标准储备液,配制成质量浓度为10 mg/L的混合标准中间液,标准工作液临用现配。用甲醇稀释同位素内标储备液,配制成质量浓度为1.00 mg/L的同位素内标混合工作液。

### 1.3 样品前处理

#### 1.3.1 酶解与样品提取

取中华鳖可食部分,放入搅拌机中制为肉糜。称取2.50 g(精确至0.01 g)肉糜试样,置于50 mL带盖离心管中,加入混合内标工作液100 μL,混匀后避光静置10 min。向试样所在离心管中加入6 mL 0.10 moL/L乙酸铵缓冲溶液(pH 4.8)和50 μL酸性磷酸酯酶,充分涡旋混匀后于37 ℃下避光恒温水浴振荡酶解2 h。取出离心管后试样冷却至室温,加入14 mL乙腈(含1%乙酸)溶液,涡旋振荡提取10 min,于4 ℃下5000 r/min离心5 min,待净化。

#### 1.3.2 样品净化

移取2 mL上清液于Wallelut Lipoclean磷脂去除小柱,自然流干后再加入0.5 mL 70%乙腈水溶液润洗柱子,收集全部流出液,40 ℃下氮吹至近干,用1.0 mL甲醇-乙腈(50∶50, v/v)溶液复溶,过0.22 μm聚四氟乙烯滤膜,待测。

### 1.4 色谱条件

色谱柱:Poroshell 120 HILIC-Z柱(100 mm×2.1 mm, 2.7 μm,美国安捷伦公司),带聚醚醚酮(PEEK)材质内衬;柱温:40 ℃;流速:0.4 mL/min;进样量:1 μL。流动相A: 0.1%(v/v)甲酸水溶液(含2 mmol/L乙酸铵);流动相B:乙腈。梯度洗脱程序:0~1.0 min, 3%A; 1.0~5.0 min, 3%A~20%A; 5.0~6.0 min, 20%A; 6.0~6.5 min, 20%A~50%A; 6.5~8.0 min, 50%A; 8.0~8.5 min, 50%A~3%A; 8.5~12 min, 3%A。

### 1.5 质谱条件

电喷雾离子源,正离子模式;扫描方式:多反应监测(MRM)模式;离子源温度:550 ℃;电喷雾电压:5500 V;雾化气(GS1)压力:345 kPa;辅助气(GS2)压力:379 kPa;气帘气(CUR)压力:172 kPa。待测药物和同位素内标的保留时间、离子对、去簇电压(DP)、碰撞能量(CE)等参数见表1。

**表1:** 10种目标物、尿苷和8种同位素内标的质谱参数

No.	Compound	Acronym	*t*_R_/min	Ion pairs (*m/z*)	DP/V	CEs/eV	IS
1	1,2,4-triazole-3-carboxamide	TCONH_2_	1.42	113.0>96.0^*^, 113.0>69.0	40	18, 35	RBV-^13^C_5_
2	lamivudine	3TC	2.29	230.1>112.1^*^, 230.1>95.0	30	23, 53	3TC-^13^C,^15^N_2_
3	imiquimod	IMQ	2.37	241.2>185.1^*^, 242.2>168.1	70	35, 45	IMQ-D_9_
4	ribavirin	RBV	3.20	245.1>113.0^*^, 245.1>96.0	30	12, 42	RBV-^13^C_5_
5	rimantadine	RMT	3.70	180.2>163.1^*^, 180.2>81.1	40	22, 30	RMT-D_4_
6	oseltamivir	OST	4.01	313.2>166.0^*^, 313.2>225.1	40	26, 15	OST-D_3_
7	amantadine	AMT	4.06	152.1>135.1^*^, 152.1>93.1	40	23, 38	AMT-D_6_
8	acyclovir	ACV	4.29	226.1>152.1^*^, 226.1>135.0	40	20, 40	ACV-D_4_
9	moroxydine	ABOB	4.65	172.1>113.0^*^, 172.1>85.0	50	28, 22	ACV-D_4_
10	oseltamivir acid	OSTA	6.13	285.2>197.1^*^, 285.2>138.1	20	15, 28	OSTA-D_3_
11	uridine	URD	2.63	245.1>113.0	30	15	-
	Lamivudine-^13^C,^15^N_2_	3TC-^13^C,^15^N_2_	2.30	233.1>115.0	30	20	-
	Imiquimod-D_9_	IMQ-D_9_	2.37	250.2>186.1	70	35	-
	Ribavirin-^13^C_5_	RBV-^13^C_5_	3.20	250.1>113.0	30	12	-
	Rimantadine-D_4_	RMT-D_4_	3.70	184.2>167.1	40	20	-
	Oseltamivir-D_3_	OST-D_3_	4.01	316.2>228.1	40	15	-
	Amantadine-D_6_	AMT-D_6_	4.06	158.2>141.1	40	23	-
	Acyclovir-D_4_	ACV-D_4_	4.29	230.1>152.1	40	20	-
	Oseltamivir acid-D_3_	OSTA-D_3_	6.13	288.2>200.0	20	15	-

* Quantitative ion. DP: declustering potential; CEs: collision energies.

### 1.6 数据处理

基质效应(matrix effect, ME)按以下公式计算^[28]^: ME=(*k*_1_/*k*_2_-1)×100%(其中*k*_1_为基质匹配标准曲线的斜率,*k*_2_为溶剂标准曲线的斜率)。绝对回收率(absolute recovery, AR)按以下公式计算^[29]^: AR=(*A/B*)×100%(其中*A*为基质加标溶液经处理后待测液目标药物的峰面积,*B*为与基质加标待测液同等浓度的纯试剂标准溶液中目标药物的峰面积)。

## 2 结果与讨论

### 2.1 质谱条件的优化

采用针泵进样对所有待测物和尿苷的质谱参数进行优化。参照文献[30]选择ESI^+^质谱模式,筛选出强度稳定且响应较高的2对离子对,并对所有离子对的DP、CE等参数进行优化,优化后的质谱参数见表1。

实验对比了利巴韦林和尿苷的二级质谱碎片信息(见图1),发现两者主要碎片均为糖苷键断裂后产生的[M-C_5_H_8_O_4_]^+^离子峰(*m/z* 113.0),由于利巴韦林和尿苷同为核苷类物质,相对分子质量接近且二级碎片一致性较高,无法在三重四极杆质谱仪上对两者进行区分,因此需要通过色谱方法进行分离。

**图 1:**
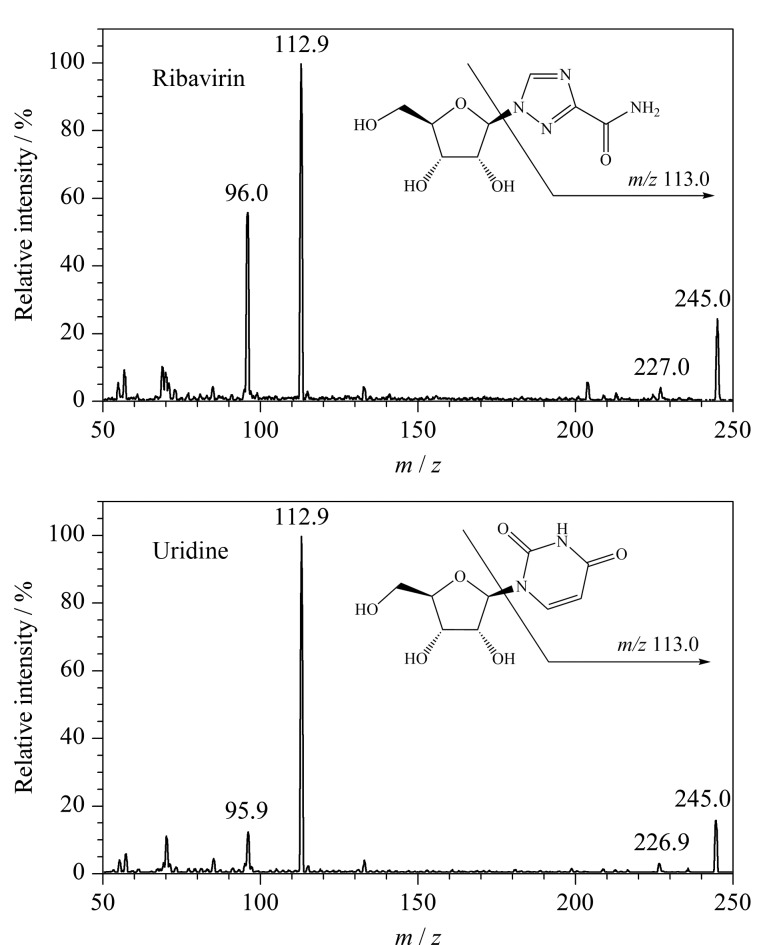
利巴韦林和尿苷的二级离子质谱图

### 2.2 色谱条件的优化

#### 2.2.1 色谱柱的选择

色谱柱的选择对其分析结果的准确度和灵敏度均会产生重要影响。实验考察了6种色谱柱对待测物质谱分析的影响,其中反相作用色谱(RPLC)条件参照Mu等^[24]^报道的方法,亲水作用色谱(hydrophilic interaction liquid chromatographic, HILIC)条件参照SN/T 4253-2015标准方法^[6]^,仪器系统稳定后重复进样3次,在获取所有待测物质谱响应值的同时考察尿苷和利巴韦林的分离效果(见图2和表2)。结果表明,HILIC模式下各待测物的质谱响应均值均强于或者接近RPLC模式,更适合于本研究中ATVs及代谢物的分析。特别是利巴韦林与其代谢物1,2,4-三氮唑-3-甲酰胺在RPLC色谱模式下保留能力弱且质谱响应较低,而HILIC模式下两者能获得较好的保留,质谱响应均值也能相应提升2~5倍。HILIC模式下Poroshell 120 HILIC-Z色谱柱上利巴韦林和尿苷的分离度(*R*_s_=4.64)高于BEH Amide色谱柱(*R*_s_=1.45),排除尿苷干扰的能力更强,因此被选为本方法的分析柱。

**图 2:**
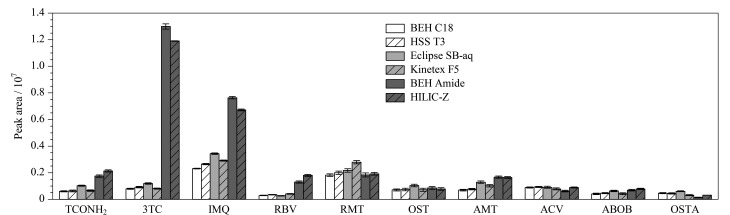
10种目标物(50 ng/mL)在不同色谱柱上的峰面积(*n*=3)

**表2:** 利巴韦林和尿苷在不同色谱柱上的分离度

No.	Brand	Model	Specification	Category	*R*_s_
1	Waters	ACQUITY UHPLC BEH C18	100 mm×2.1 mm, 1.7 μm	RPLC	4.15
2	Waters	ACQUITY UHPLC HSS T3	100 mm×2.1 mm, 1.8 μm	RPLC	4.69
3	Agilent	ZORBAX Eclipse SB-aq	100 mm×2.1 mm, 1.8 μm	RPLC	5.65
4	Phenomenex	Kinetex F5	100 mm×2.1 mm, 1.7 μm	RPLC	5.15
5	Waters	ACQUITY UHPLC BEH Amide	100 mm×2.1 mm, 1.7 μm	HILIC	1.45
6	Agilent	Poroshell 120 HILIC-Z	100 mm×2.1 mm, 2.7 μm	HILIC	4.64

#### 2.2.2 流动相的优化

HILIC色谱模式通常采用乙腈流动相体系,流动相中缓冲盐的浓度会显著影响化合物的峰形、保留时间和灵敏度。为此,实验进一步考察了不同浓度乙酸铵溶液(0.5、1、2、5、10、20 mmol/L,均含0.1%甲酸)-乙腈流动相体系对待测物峰面积的影响(见图3)。结果表明,随着乙酸铵溶液浓度的上升,10种待测物峰面积呈现下降趋势,下降幅度为10.7%~34%(乙酸铵浓度0.5 mmol/L与20 mmol/L时对比)。此外,当乙酸铵溶液浓度为2 mmol/L及以上时各待测物峰形能保持稳定,低于此浓度后金刚烷胺、吗啉胍等药物出峰时间易出现飘移或出现峰分叉,这与Douillet等^[31]^的研究结果相一致。

**图 3:**
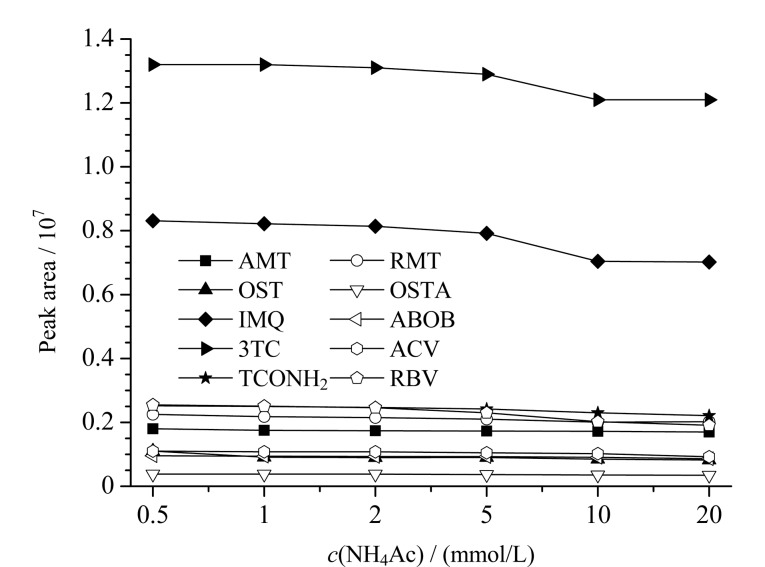
不同浓度乙酸铵溶液对10种目标物峰面积的影响(*n*=3, 50 ng/mL)

综合考虑,本研究采用2 mmol/L乙酸铵溶液(含0.1%甲酸)作为流动相,此时10种待测物的响应相比乙酸铵溶液浓度为20 mmol/L时能提高8.1%~29%。进一步优化梯度程序等,得到1.4节的色谱条件,此条件下10种抗病毒药物及代谢物色谱峰形良好,同时利巴韦林和尿苷仍能确保基线分离排除干扰(见图4)。

**图 4:**
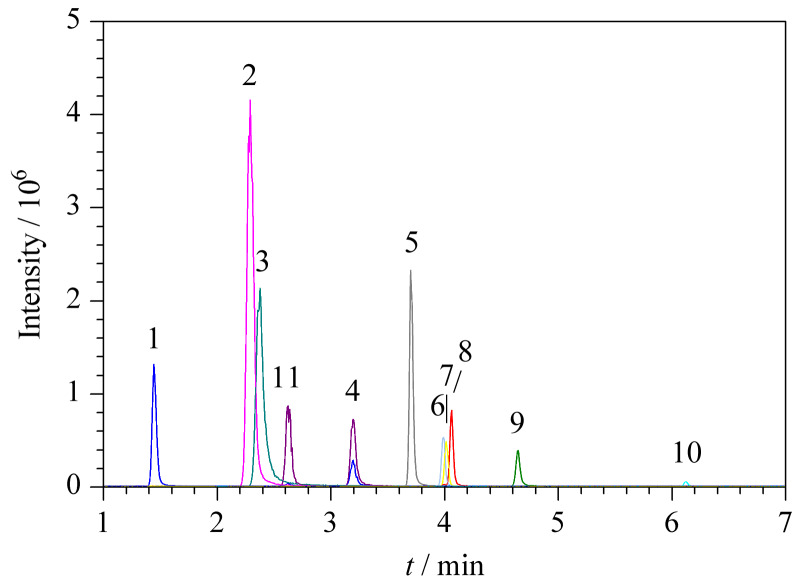
尿苷与10种目标物的提取离子色谱图

### 2.3 前处理条件优化

#### 2.3.1 前处理方式的选择

实验前期验证了报道的QuEChERS法^[24]^和吸附-洗脱模式固相萃取法^[21]^对10种待测物的提取和净化效果。结果表明,QuEChERS法中*N*-丙基乙二胺(PSA)吸附剂对代谢物奥司他韦酸存在强吸附,吸附-洗脱模式固相萃取法中的混合型阳离子交换(MCX)小柱对强极性药物利巴韦林等无法有效保留,均存在适用性问题。此外,利巴韦林在生物体内多以磷酸酯形式存在,需要酶解为游离态以确保分析的准确性^[5]^。因此本实验先采用乙酸铵缓冲溶液(0.1 mol/L, pH 4.8)分散样品基质后酶解,再使用极性适中的乙腈(含1%乙酸)试剂^[19,24,31]^进行二次提取,最后采用通过式固相萃取方式净化样品。上述前处理方式既保证了样品的酶解需求,也兼顾了不同极性待测物的提取需要,同时无需溶剂置换,提取液可直接采用通过式固相萃取净化。

#### 2.3.2 前处理条件的确定

提取液中乙腈含量会同时影响待测物的提取效率和后续的净化效果,需综合考察后再确认其最优比例。在称样量和提取液总量固定为2.50 g和20 mL的条件下,考察了不同乙腈(含1%乙酸)比例提取液(分别为10%、20%、30%、40%、50%、60%、70%、80%和90%)对10种待测物提取率的影响(见图5)。结果表明,当提取液乙腈(含1%乙酸)比例低于50%时,咪喹莫特等部分极性较弱药物的提取率出现下降并低于70%,当提取液乙腈(含1%乙酸)比例达到90%时,利巴韦林等部分强极性药物的提取率会出现下降并低于80%。提取液中乙腈(含1%乙酸)比例为50%~80%时能够兼顾绝大多数待测物的提取效率,此时10种待测物的平均提取率为81%~94%。

**图 5:**
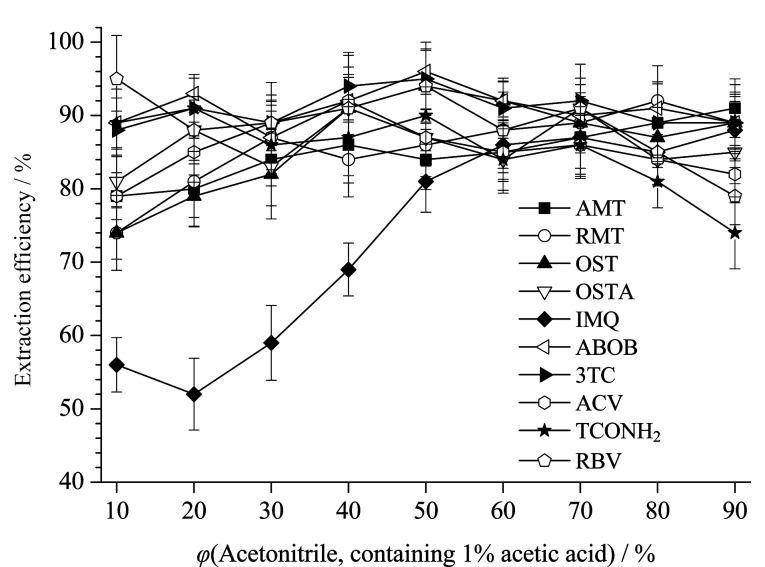
提取液中乙腈比例对10种目标物提取率的影响(*n*=3)

在提取率试验结果基础上,比较了不同乙腈(含1%乙酸)比例提取液在不同通过式固相萃取小柱上对所有待测物的净化效果。按1.3.1节方法获取中华鳖阴性样品基质溶液,添加待测物后分别通过Oasis PRIME HLB小柱(150 mg/3 mL,美国沃特世公司)、Captiva EMR-Lipid小柱(300 mg/3 mL,美国安捷伦公司)和Wallelut Lipoclean小柱净化,以绝对回收率评价净化效果。由图6可知Captiva EMR-Lipid小柱和Oasis PRIME HLB小柱分别对吗啉胍和阿昔洛韦存在吸附,净化后的绝对回收率显著低于未净化组。Wallelut Lipoclean小柱整体净化效果最优,在利巴韦林、阿昔洛韦、咪喹莫特等药物上具有最高的绝对回收率,被选为方法的净化小柱。按照文献[32]报道,通过式固相萃取柱的上样溶液乙腈比例通常为70%~85%,此时可以较好地平衡净化效果与待测物的回收率。本实验提取液中乙腈(含1%乙酸)比例选择为70%,此时大部分待测物的绝对回收率最高(范围为75%~95%)。综上,本实验最终采用6 mL 0.10 moL/L乙酸铵缓冲溶液加14 mL乙腈(含1%乙酸)的提取模式,此时提取液中乙腈(含1%乙酸)比例为70%。

**图 6:**
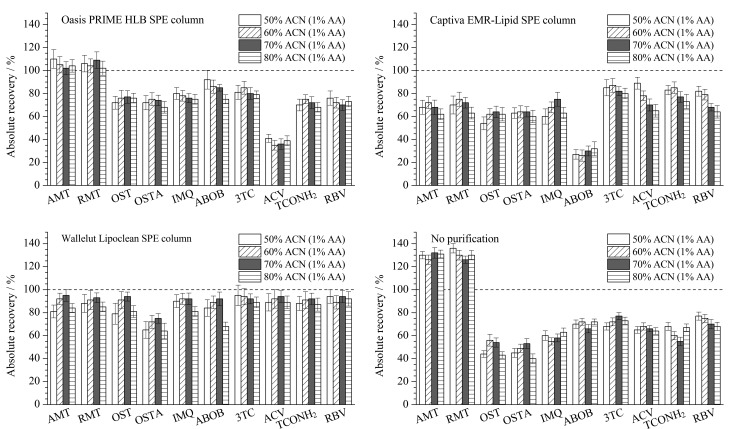
不同固相萃取柱对10种目标物绝对回收率的影响(*n*=3)

### 2.4 基质效应

质谱分析中样品基质会影响目标物的离子化程度,导致基质效应的出现,而较强基质效应的存在会影响质谱分析的准确性^[27]^。经考察,中华鳖基质下金刚烷胺和金刚乙胺表现出弱效应的基质增强作用,|ME|分别为6.3%和5.6%,其余待测物均存在弱效应的基质抑制作用,|ME|范围为5.5%~16%。为了校正基质效应,本实验采用同位素稀释内标法进行定量分析。

### 2.5 检出限、定量限与线性范围

采用内标法绘制标准曲线,10种待测物在线性范围内均具有良好的线性关系,相关系数(*r*^2^)均大于0.993。按照标准GB/T 27417-2017,以空白标准偏差法确定方法检出限(MDL)和定量限(MQL), 10种待测物的MDL范围为0.05~1.1 μg/kg, MQL范围为0.18~3.8 μg/kg(见表3)。现有行业标准^[5,6]^中金刚烷胺、金刚乙胺、利巴韦林、咪喹莫特、吗啉胍和奥司他韦的MQL均为1.0 μg/kg,阿昔洛韦为2.0 μg/kg,本方法对应的ATVs灵敏度均接近或优于标准方法。

**表3:** 10种目标物的线性范围、线性方程、相关系数、检出限和定量限

Compound	Linear range/(μg/L)	Linear equation	*r*^2^	MDL/(μg/kg)	MQL/(μg/kg)
TCONH_2_	1-100	*y*=0.0916*x*+0.0129	0.998	0.26	0.88
3TC	0.2-20	*y*=0.0174*x*-0.00010	0.997	0.05	0.18
IMQ	0.25-25	*y*=0.0624*x*-0.00313	0.998	0.06	0.21
RBV	1-100	*y*=0.0383*x*-0.000642	0.995	0.29	0.98
RMT	0.5-50	*y*=0.0240*x*+0.000732	0.996	0.12	0.41
OST	0.5-50	*y*=0.0243*x*+0.000556	0.994	0.15	0.48
AMT	0.5-50	*y*=0.0255*x*+0.0023	0.995	0.14	0.46
ACV	0.5-50	*y*=0.0243*x*+0.00134	0.998	0.14	0.46
ABOB	0.5-50	*y*=0.0107*x*+0.00181	0.995	0.15	0.50
OSTA	4-400	*y*=0.0315*x*-0.000583	0.995	1.1	3.8

*y*: peak area ratio of the analyte to the internal standard; *x*: mass concentration, μg/L.

### 2.6 回收率与精密度

选取空白中华鳖样品,按低、中、高3个水平添加目标物,每个水平重复测定6次,计算回收率和RSD值。由表4可知,10种待测物的加标回收率范围为82.5%~103%,相对标准偏差(RSD)为3.11%~12.1%。

**表4:** 10种目标物在鳖样品中的加标回收率和相对标准偏差(*n*=6)

Compound	Spiked level/(μg/kg)	Recovery/%	RSD/%
TCONH_2_	1	98.6	9.18
	2	103	7.21
	10	97.9	5.08
3TC	0.2	94.1	9.23
	0.4	92.9	5.49
	2	95.2	3.21
IMQ	0.25	94.1	9.23
	0.5	92.9	5.49
	2.5	95.2	3.21
RBV	1	91.7	5.98
	2	93.9	6.33
	10	95.0	4.62
RMT	0.5	87.2	11.2
	1	92.6	6.57
	5	93.7	5.72
OST	0.5	90.7	7.50
	1	95.2	4.30
	5	97.2	3.11
AMT	0.5	90.4	9.20
	1	94.6	7.91
	5	95.8	6.66
ACV	0.5	95.1	6.88
	1	96.1	4.89
	5	99.5	7.79
ABOB	0.5	82.5	9.17
	1	84.9	6.28
	5	83.4	4.58
OSTA	4	88.6	12.1
	8	91.2	9.86
	40	90.7	6.16

### 2.7 实际样品分析

采用本方法测定了地方水产养殖场中67批次中华鳖样品,其中3批次样品检出利巴韦林,含量分别为6.02、46.2和302 μg/kg。此外,利巴韦林含量最高的样品中还检出利巴韦林生物代谢物1,2,4-三氮唑-3-甲酰胺残留,含量为31.2 μg/kg。采用行业标准^[5]^对利巴韦林阳性样品进行对比检测,检测结果分别为5.76、51.2和312 μg/kg,与本方法检测结果一致性较好。

## 3 结论

本研究建立了中华鳖中10种抗病毒药物及代谢物的通过式固相萃取-超高效液相色谱-串联质谱分析方法。该方法前处理简单,特异性强,灵敏度高,适用于中华鳖中多种抗病毒药物及代谢物的快速精准检测分析。研究结果表明,中华鳖中存在利巴韦林残留风险,建议进一步开展中华鳖中利巴韦林的药代动力学、组织分布规律和迁移转化规律研究,为风险溯源和防控提供理论依据和指导。
